# Fabry Disease: Insights into Pathophysiology and Novel Therapeutic Strategies

**DOI:** 10.3390/biomedicines13030624

**Published:** 2025-03-04

**Authors:** Sophie Elizabeth Thompson, Ashwin Roy, Tarekegn Geberhiwot, Katja Gehmlich, Richard Paul Steeds

**Affiliations:** 1 Department of Cardiovascular Sciences, School of Medical Sciences, College of Medicine and Health, University of Birmingham, Birmingham B15 2TT, UK; 2 Department of Cardiology, Queen Elizabeth Hospital, University Hospital Birmingham NHS Foundation Trust, Birmingham B15 2TH, UK; 3 Department of Diabetes, Endocrinology and Metabolism, University Hospital Birmingham NHS Foundation Trust, Birmingham B15 2TH, UK; 4 Institute of Metabolism and System Sciences, School of Medical Sciences, College of Medicine and Health, University of Birmingham, Birmingham B15 2TT, UK; 5 Division of Cardiovascular Medicine, Radcliffe Department of Medicine and British Heart Foundation Centre of Research Excellence Oxford, University of Oxford, Oxford OX1 2JD, UK

**Keywords:** Fabry disease, inflammation, heart failure, chronic kidney disease

## Abstract

Fabry disease (FD) is an X-linked lysosomal storage disorder characterized by deficiency of α-galactosidase A (α-GalA), leading to the accumulation of glycosphingolipids and multi-organ dysfunction, particularly affecting the cardiovascular and renal systems. Disease-modifying treatments such as enzyme replacement therapy (ERT) and oral chaperone therapy (OCT) have limited efficacy, particularly in advanced disease, prompting a need for innovative therapeutic approaches targeting underlying molecular mechanisms beyond glycosphingolipid storage alone. Recent insights into the pathophysiology of FD highlights chronic inflammation and mitochondrial, lysosomal, and endothelial dysfunction as key mediators of disease progression. Adjunctive therapies such as sodium-glucose cotransporter-2 (SGLT2) inhibitors, glucagon-like peptide-1 (GLP-1) agonists, and mineralocorticoid receptor antagonists (MRAs) demonstrate significant cardiovascular and renal benefits in conditions including heart failure and chronic kidney disease. These drugs also modulate pathways involved in the pathophysiology of FD, such as autophagy, oxidative stress, and pro-inflammatory cytokine signaling. While theoretical foundations support their utility, dedicated trials are necessary to confirm efficacy in the FD-specific population. This narrative review highlights the importance of expanding therapeutic strategies in FD, advocating for a multi-faceted approach involving evidence-based adjunctive treatments to improve outcomes. Tailored research focusing on diverse FD phenotypes, including females and non-classical variants of disease, will be critical to advancing care and improving outcomes in this complex disorder.

## 1. Introduction

Fabry disease (FD) is an X-linked lysosomal storage disorder resulting from a mutation in the GLA gene, which leads to deficiency or reduced activity of the enzyme α galactosidase A (α-GalA). Deficiency of this enzyme produces lysosomal accumulation of glycosphingolipids, namely, globotriasylceramide (Gb3) and globotriaosylsphingosine (lyso-Gb3) [1]. Accumulation affects multiple organs and leads to progressive cardiomyopathy, renal impairment, and cerebrovascular events. Prevalence of FD is estimated at 1 in 40,000 to 1 in 170,000 [2]. Men are typically affected at a younger age compared to women, owing to the X-linked nature of the condition. However, manifestations of FD in women are heterogenous due to random inactivation of X chromosomes (lyonization), meaning the spectrum of disease in women ranges from mild disease to a severe phenotype like that seen in males [3].

The ‘classical variant’ of FD is characterized by low or absent α-GalA activity, resulting in severe multi-system glycosphingolipid accumulation, manifesting in childhood or adolescence. In classical FD, variants in the GLA gene result in truncated or complete/near complete enzyme deficiency. Cardiomyopathy may be evident by the second decade, along with renal impairment that may progress to end-stage renal failure requiring dialysis or transplantation [4]. In patients ≥ 40 years, it is estimated that 41% of males and 20% of females have at least stage 3 chronic kidney disease (CKD) [5]. Cerebrovascular events including transient ischemic attack and stroke also occur at a young age and may cause significant disability and death [6]. In the milder ‘non-classical variant’ of FD, residual α-GalA activity is present. Presentation is often later, in the fifth decade of life, and single organ (usually cardiac) with left ventricular hypertrophy (LVH), diastolic dysfunction, arrhythmia, conduction disease, and coronary artery disease [4]. Cardiomyopathy is the leading cause of death in patients with FD [7,8]. It is thought that around 1.6% of patients diagnosed with unexplained LVH have FD [9].

**Figure 1:**
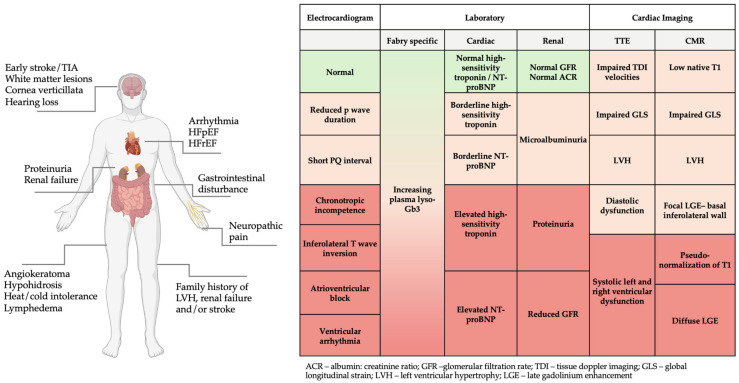
Red flag clinical features, electrocardiogram, laboratory, and imaging findings in FD. Figure created in BioRender (https://biorender.com/) [accessed on 31 January 2025].

Diagnosis of FD is often delayed, with patients reporting symptoms for many years before a formal diagnosis is made [10]. Unfortunately, the diagnosis is often only considered in the later stages of disease once LVH, cardiomyopathy, and renal failure ensues. FD should be suspected in patients with red-flag clinical features or abnormal laboratory and/or imaging investigations (Figure 1), particularly in the presence of a positive family history. Once diagnosed, family screening should be offered to allow early detection of related cases. While scoring systems such as the Mainz Severity Score Index (MSSI) and disease severity scoring index (DS3) help to quantify disease severity and risk of progression, there are no widely adopted risk prediction tools to trigger genetic testing in FD. Recently, Lobel et al. (2024) developed the FDrisk tool, a statistical risk prediction model developed using electronic health records of patients with FD. Although this tool performed well in predicting individual risk for FD based on symptoms and signs, it is yet to be established in clinical practice [11].

The mainstay of treatment in FD is enzyme replacement therapy (ERT), and more recently, oral chaperone therapy (OCT). In cardiovascular terms, these aim to slow progression of LVH, prevent the development of fibrosis with resultant heart failure, and reduce morbidity and mortality [12]. Although evidence for impact on renal function is stronger, there remain concerns whether disease-specific therapy sufficiently impacts the cardiovascular manifestations of FD [13]. It is clearer that once renal disease is established, suggested by the presence of proteinuria and glomerulosclerosis, ERT has limited efficacy [14]. Similarly, ERT has shown limited efficacy when LVH/fibrosis is established [15]. This suggests that there is a point beyond which other secondary mediators of disease are more important than primary enzyme deficiency and novel therapeutic strategies are needed to address these mechanisms. Although many adjunctive therapies that have proven utility in cardiac and renal disease of other etiologies have been used in FD patients, there is a dearth of evidence on how these may have an effect in FD, and even less data from controlled trials. The aim of this narrative review is to explore the pathophysiology underpinning cardiovascular and renal manifestations of FD and discuss novel therapeutics relevant to these mechanisms of disease that may be worthy of greater study.

## 2. Pathophysiology

Cardiomyopathy is the leading cause of death in patients with FD [7,8]. Gb3 is thought to accumulate in all cardiac cell types, including endothelial and smooth muscle cells, fibroblasts, and cardiac myocytes, disrupting cellular function. Gb3 accumulation in myocardium leads to LVH, diastolic dysfunction, and fibrosis, including of conduction tissue giving rise to the high burden of arrhythmia [16]. Transthoracic echocardiography (TTE) is the first line imaging modality to investigate suspected cardiovascular disease and is widely used in the assessment and follow-up of Fabry cardiomyopathy, as it is readily available in the clinic. Techniques such as tissue doppler imaging (TDI) and speckle tracking global longitudinal strain (GLS) hold promise in detecting early stages of disease [17]. Cardiac magnetic resonance imaging (CMR), although not as readily available, is superior to TTE in the diagnosis and surveillance of FD, providing more detailed anatomical and structural assessment, along with novel mapping techniques and tissue assessment with gadolinium contrast. CMR can identify pathological change in mild or subclinical phases of disease prior to many identifiable changes on TTE [18]. CMR can also reliably distinguish FD from other causes of LVH using techniques such as T1 and T2 mapping, which quantifies longitudinal and transverse relaxation times [19].

**Figure 2:**
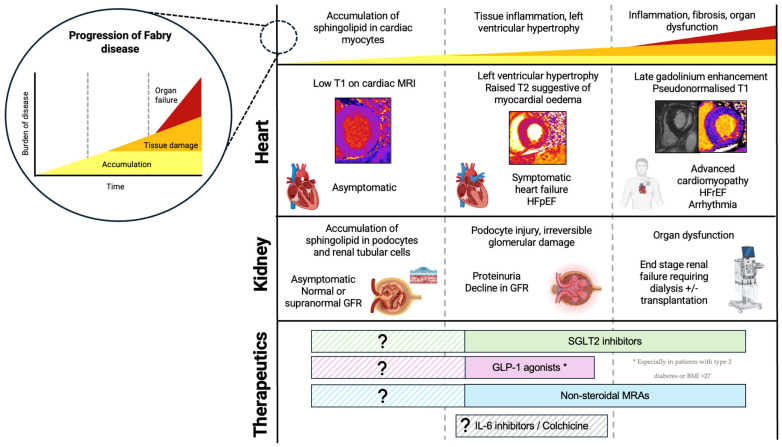
Progression of FD, including cellular and histological changes, clinical features, and cardiac MRI findings. Novel therapeutics and the proposed time at which they may be useful in FD. Figure created in BioRender (https://biorender.com/) [accessed on 31 January 2025].

A three-stage model of cardiomyopathy progression in FD has been proposed (Figure 2) [20]. Firstly, accumulation of glycosphingolipid in the myocardium begins, often in childhood, occurring earlier and progressing faster in males. On CMR, sphingolipid accumulation manifests as low native T1 values, and this is thought to precede the typical cardiac phenotype, detectable in the absence of other features of cardiomyopathy such as LVH [19,21]. Early functional changes also occur, such as impairment in global longitudinal strain (GLS). GLS worsens as native T1 values reduce, and this precedes the hypertrophic phase of disease [22]. Impaired GLS, particularly inferiorly, is also one of the earliest features of Fabry cardiomyopathy identifiable on TTE [23]. Furthermore, Pieroni et al. (2003) demonstrated that reduction in systolic (s′) velocity and early myocardial relaxation velocity (e′) were detectable even before the development of LVH, and reductions in TDI velocities were proportional to sphingolipid accumulation on endomyocardial biopsy [24]. This early accumulation stage is thought to be subclinical; however, emerging evidence shows that FD patients exhibit impaired exercise capacity even in the earliest stages of disease, suggesting cardiomyopathy may begin developing even before detectable changes on imaging [25]. In the second stage of disease, LVH develops, often quicker and more extreme in males than females. This phase of disease can be identified on TTE by an increase in LV wall thickness, with LVH being the most reported TTE finding in FD. It remains difficult to distinguish Fabry cardiomyopathy from other causes of LVH on TTE alone. The ‘binary sign’, a hyperechogenic endocardial surface adjacent to a hypoechogenic subendocardial layer visible on TTE reflecting glycosphingolipid accumulation, was once thought to help distinguish FD from other causes of hypertrophic cardiomyopathy; however, this is no longer deemed specific to FD [26]. Diastolic dysfunction may also be apparent on TTE, with increased left atrial volumes and worsening TDI velocities over time [27,28]. Changes in atrial dimensions and impaired atrial function (impaired left atrial GLS, reduced left atrial ejection fraction) are also evident, and may not be simply a consequence of elevated LV pressures, but rather intrinsic atrial myopathy suggestive of diffuse disease [27,28,29]. As cardiomyopathy progresses, fibrosis suggestive of myocyte death develops. Typically, on CMR, late gadolinium enhancement (LGE) is demonstrated in the basal inferolateral wall [30]. Interestingly in women, LGE may manifest prior to LVH and the mechanism behind this is poorly understood. Native T1 values which were low in early disease ‘pseudo-normalize’ in areas of myocardial fibrosis. Elevated T2 values on CMR are also found in areas of fibrosis, indicating myocardial oedema and inflammation [31]. T2 values correspond closely with release of high-sensitivity troponin detected in the blood [32]. Inflammation is likely to play a primary role in the development of LVH, LGE, and fibrosis, and can be demonstrated on PET/MR imaging with fluorine-18 fluorodeoxyglucose positron emission tomography ((18)F-FDG PET) cardiac uptake [33]. Further supporting this, immune-mediated myocarditis is detectable on endomyocardial biopsies of patients with FD at varying degrees of LVH and correlates with disease severity [34]. In late stages of disease, clinical features of heart failure and systolic impairment may become apparent. Advanced cardiomyopathy is characterized by impaired left and right ventricular function; however, even in the most advanced stages of cardiomyopathy, only a small proportion exhibit a significant decline in LVEF [27,28].

Gb3 also accumulates in all renal cell types including interstitial, tubular, endothelial, and glomerular cells [35]. Structural changes on histology include glomerular sclerosis, tubular atrophy, and interstitial fibrosis [35]. Podocyte damage is important in the pathogenesis of FD nephropathy and occurs early [36]. Podocytes become hypertrophic and have a characteristic vacuolated cytoplasm, leading to irreversible glomerulosclerosis and nephron injury. Accumulation of Gb3 in tubular cells may also contribute to renal dysfunction [37]. Microalbuminuria is a sensitive marker of early renal dysfunction and hyperfiltration may be apparent in initial stages [5,38]. Proteinuria then ensues around the second to third decade of life prior to decline in glomerular filtration rate (GFR) [39], and the degree of proteinuria correlates with rate of progression of nephropathy [40]. Renal biopsy can assess the degree of glomerulosclerosis and interstitial damage in patients with proteinuria. However, histology can also identify the degree of Gb3 deposition in podocytes, which is significant in women who may show signs of Gb3 accumulation prior to the development of clinically detectable renal disease. It has been suggested that ERT might be considered based on podocyte storage of Gb3 alone as a marker of organ involvement in FD, although this is not routine practice and further studies are needed [2].

## 3. Molecular Mechanisms

**Figure 3:**
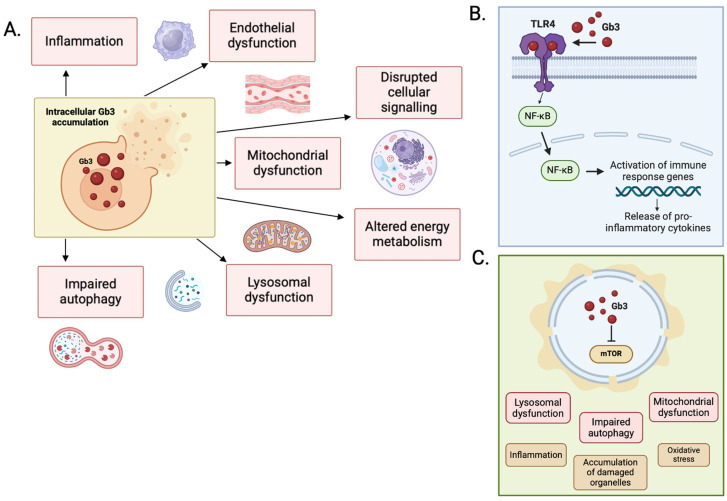
(**A**) Primary mechanisms of disease in FD. (**B**) Secondary pathways implicated in the pathophysiology of FD. Activation of the TLR-4/NF-κB pathway by Gb3 stimulates the release of pro-inflammatory cytokines. (**C**) Accumulation of sphingolipid disrupts the function of mTOR, with downstream effects on autophagy, lysosomal, and mitochondrial function. Figure created in BioRender (https://biorender.com/) [accessed on 31 January 2025].

The mechanical effects of accumulation of Gb3 alone are not enough to explain the degree of LVH seen in the heart of patients with end-stage FD. In one study of FD patients with severe LVH at autopsy, Gb3 accumulation contributed to less than 5% total LV mass [41]. The exact mechanism by which Gb3 accumulation triggers LVH is not understood, but it is likely that this is a combination of the impact of intracellular accumulation on cellular signaling pathways, increased autophagy and apoptosis, lysosomal dysfunction, mitochondrial dysfunction and altered energy metabolism, and extracellular overspill triggering endothelial cell dysfunction and pro-inflammatory signaling (Figure 3).

### 3.1. Lysosomal Dysfunction

Lysosomes function as a regulatory organelle, responsible for degrading both intracellular and extracellular substrates by endocytosis, phagocytosis, and autophagy. Lysosomes contain numerous hydrolytic enzymes that degrade proteins, polysaccharides, lipids, and DNA. Lack of the enzyme α-GalA limits the breakdown of glycolipids and glycoproteins, which impairs the ability of the lysosome to exert its autophagic clearance functions, causing cellular damage.

### 3.2. Mitochondrial Dysfunction and Energy Metabolism

An important function of the lysosome is mitophagy, that is, the removal of damaged mitochondria through autophagy to preserve cellular mitochondrial function. Lysosomal dysfunction and mitochondrial dysfunction are closely related [42]. Mitochondrial activity is essential for cardiac energy metabolism and the optimal functioning of cardiac myocytes. In vivo studies have demonstrated impaired cardiac energy metabolism in patients with FD, evidenced by reduced phosphocreatine to adenosine triphosphate ratio, which improves with ERT [43]. Interestingly, the positive changes in energy metabolism following ERT preceded the regression of LVH [43]. One mechanism that may account for impaired energy metabolism involves the mammalian target of rapamycin kinase (mTOR). mTOR is found on the surface of lysosomes and mitochondria, functioning to inhibit lysosomal fusion and autophagy and promote protein synthesis and cell growth [44,45]. AMP-activated protein kinase (AMPK) inhibits mTOR activity in response to reduced ATP levels [46]. Impairment in mTOR activity has been demonstrated in fibroblasts and podocytes of patients with FD, and this may in part be explained by accumulation of sphingolipids in lysosomes, disrupting mTOR’s activity [47]. Loss of mTOR activity, which negatively modulates autophagy, may lead to the accumulation of damaged organelles, increasing oxidative stress, cellular dysfunction and ultimately, organ dysfunction [48].

### 3.3. Chronic Inflammation and the Immune System

Arguably, more important that the storage element of FD itself, is the inflammatory cascade that is triggered by damaged cells and dysregulated cellular functions, mediated by the innate and adaptive immune systems.

Cells of the innate immune system, including macrophages, dendritic cells, and neutrophils, respond to damage-associated molecular patterns (DAMPs) via toll-like receptors (TLRs). TLR-4 specifically has been implicated in FD, as TLR-4 activates the NF-kB pathway leading to the release of pro-inflammatory cytokines including TNF-α, IL-1β, and IL-6 amongst others. TLR-4 recognizes molecules exposed during cellular injury but has also been shown to activate in response to lyso-Gb3 seen in FD [49]. Blood samples from 29 FD patients demonstrated significant increases in IL-6 and IL-1β compared to health controls. Production of these pro-inflammatory cytokines were abolished in vitro when using TLR-4 blocking antibodies [50]. Inflammatory cells including macrophages, neutrophils, and mast cells are mobilized in response to cellular stress and pro-inflammatory cytokines. Macrophage-related markers including CD68, CD163, and CD45 have been identified on histology in the hearts and kidneys of FD patients [51,52,53]. Monocyte chemoattractant protein-1 (MCP-1) is an important chemokine in macrophage recruitment. Levels of MCP-1 along with pro-inflammatory cytokines such as TNF-α are increased in the serum of patients with FD and decrease over time in response to ERT [54].

The adaptive immune system, which responds to specific pathogens marked as foreign, is thought to play a lesser role in FD, although there does seem to be an association between FD and autoimmunity, so it is likely that the role is not fully understood [55].

Inflammation in FD is chronic and unrelenting, evidenced by the persistence of macrophage-related markers on endomyocardial biopsy compared to other inflammatory cardiomyopathies such as myocarditis [51]. This is unsurprising given that the presence of glycosphingolipid provides a continuous underlying trigger for inflammation. Plasma levels of inflammatory and remodeling biomarkers are higher in patients with LVH and MRI evidence of fibrosis [56]. Chronic inflammation leads to the proliferation of fibroblasts, extracellular remodeling, and collagen deposition, with the primary aim of tissue repair; however, unchecked, this may lead to organ fibrosis. Clinically, this gives rise to a phenotype of progressive renal impairment due to glomerular fibrosis, heart failure with preserved ejection fraction (HFpEF), and conduction disease.

### 3.4. Endothelial Dysfunction

Vascular dysfunction is thought to be another important mechanism contributing to the pathophysiology and multi-system clinical manifestations seen in FD. Patients with FD have a higher incidence of intravascular thrombosis and atherosclerosis, and endothelial dysfunction likely contributes to this [57,58]. As demonstrated in many other cell types, Gb3 also accumulates in endothelial and smooth muscle cells [59]. Histologically, patients with FD show increased intima media thickness in major blood vessels, probably secondary to smooth muscle proliferation stimulated by lyso-Gb3 [60,61].

It is hypothesized that lower levels of enzymatic activity and increased lysosomal storage of Gb3 directly cause vascular damage, evidenced by the increase in pro-angiogenic markers in the serum. Vascular endothelial cells cultured with Gb3 demonstrated increased reactive oxygen species (ROS) production and expression of adhesion molecules that recruit inflammatory cells [62]. Vascular endothelial growth factor A (VEGF-A) is a pro-angiogenic factor expressed by endothelial cells, promoting endothelial cell proliferation and increasing vascular permeability. It is over-expressed in response to hypoxia and oxidative stress [63]. High VEGF-A levels have been demonstrated in patients with FD and levels correlate with low levels of α -GalA activity and severe organ involvement [64].

## 4. Disease-Modifying Treatment

ERT has been the mainstay of treatment for FD since the early 2000s. More recently, OCT has become commonplace in the management of FD, whilst substrate reduction therapy and gene therapy are subject to ongoing clinical trials.

### 4.1. Enzyme Replacement and Chaperone Therapies:

**Figure 4:**
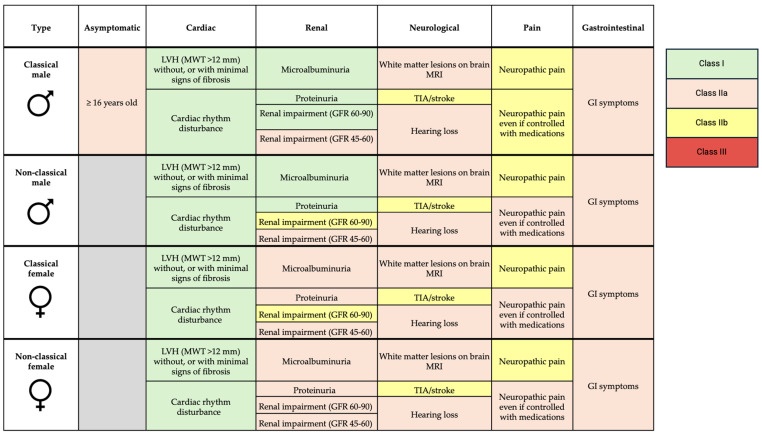
Indications for enzyme replacement therapy in FD. LVH—left ventricular hypertrophy; MWT—maximal wall thickness; GFR—glomerular filtration rate; MRI—magnetic resonance imaging; TIA—transient ischemic attack; GI—gastrointestinal.

ERT and OCT in FD are the only well-established treatment currently available for managing FD. ERT formulations available include agalsidase alfa, agalsidase beta, and more recently, pegunigalsidase alfa. The cost of ERT is estimated at around £170,000 per year and is usually delivered by intravenous infusion every 2 weeks [65]. Indications for enzyme replacement therapy are summarized in Figure 4.

ERT is recommended for patients with cardiac involvement, evidenced by hypertrophy (maximal wall thickness > 12 mm), without significant fibrosis, or in those with cardiac rhythm disturbance (Class I recommendation) [2]. ERT is thought to slow progression of LVH and cardiac dysfunction. While a small, randomized trial (*n* = 15) and observational data suggest that ERT reduces LV mass and progression of LVH, these benefits only seem to be applicable to those who already have LVH [66,67,68,69,70]. Data are conflicting, as other studies found no difference in progression with ERT [71,72]. Clinical trials have not shown improvements in LVEF with ERT despite some observational data suggesting otherwise [73,74]. In terms of clinical outcomes, a double-blind placebo randomized controlled trial of agalsidase beta in 82 patients showed no significant reduction in time to composite clinical outcome of renal, cardiac, cerebrovascular events or death, and no reduction in time to adverse cardiovascular event [75]. Better outcomes are reported with FD therapies the earlier they are initiated, but those aged >40 years are more likely to show disease progression despite treatment [76]. ERT has no role in advanced cardiomyopathy with extensive fibrosis, as it is ineffective once fibrosis is established [77]. While earlier treatment is generally better, ERT is not currently recommended in patients with evidence of myocardial sphingolipid accumulation (low T1 values on CMR) in the absence of other features of cardiomyopathy. T1 mapping is a relatively new technique, and trials are needed to assess whether ERT is efficacious at the accumulation phase of disease.

In patients with renal involvement, ERT initiation is recommended in the presence of microalbuminuria, proteinuria, and reduced GFR, depending on sex and type of FD. Small, randomized trials show stabilization, or slight improvement in GFR in patients treated with ERT [66,78]. Patients receiving ERT sooner after onset of proteinuria, and lower levels of proteinuria at the time of initiation, have more stable renal function [79,80].

OCT (migalastat) is delivered by oral tablet once daily and costs around £210,000 per year per patient [81]. OCT works by helping GLA to fold, thereby increasing enzyme activity and protein expression in those who have residual enzyme activity; however, it is only suitable for patients with an amenable genetic variant (approx. 30–35%), which are most commonly, but not always missense mutations [82]. The commonest genetic variant causing a cardiac phenotype of FD in the UK (N215S) is an amenable variant. OCT is a suitable alternative to ERT in select patients, but may also be chosen as a first-line agent given its advantage of an oral daily tablet rather than a resource intensive bi-weekly infusion. Evidence of impact on clinical outcomes is limited. One double-blind randomized trial showed a small but non-significant reduction in LV mass with migalastat versus placebo at 6 months, although at 24-months of open-label extension, LV mass did decrease significantly from baseline [83]. A direct comparison trial of migalastat versus ERT suggested superiority of migalastat in terms of reduction in LV mass, although clinical events were too infrequent in the small study population to draw conclusions about efficacy [84].

The evidence for ERT and OCT is limited and conflicting, with few RCTs and small numbers of patients, limiting statistical power and generalizability. Most data come from observational studies, which are inherently subject to confounders such as differences in disease stage in treated versus untreated patients; therefore, outcomes may reflect disease progression rather than ERT inefficacy. Furthermore, most studies have focused predominantly on males with the classical form of the disease, limiting applicability to females and non-classical phenotypes, especially as females tend to follow a different disease course, with LGE preceding LVH in many. As a result, the current indication criteria for initiating ERT may not fully capture the needs of females or those with non-classical FD, and the lack of representation of these groups in research limits the generalizability of treatment guidelines and consensus recommendations.

### 4.2. Substrate Reduction Therapy and Gene Therapy

The concept of substrate reduction therapy (SRT) in lysosomal storage disorders was first suggested in 1996 to treat Gaucher disease [85]. Substrate reduction therapies (Ibiglustat/Venglustat^®^, Lucerastat^®^) are currently being evaluated in clinical trials. The aim of SRT is to reduce production of Gb3 and lyso-Gb3 by inhibiting the glucosylceramide synthase enzyme involved in production of glycosphingolipid, reducing accumulation and thereby progression of disease. Although pre-clinical studies have demonstrated effective inhibition of glucosylceramide synthase and reduced substrate accumulation, clinical trials have failed to show improvement in primary clinical endpoints. A phase 2 clinical trial of Venglustat in 11 patients with FD did not show significant reduction in Gb3 accumulation after 6 months; however, in an extension of the study up to 3 years, there was a reduction in Gb3 accumulation and apparent disease stabilization [86]. In a larger phase 3 clinical trial of Lucerastat including 118 adults with FD, oral SRT reduced serum Gb3 levels by almost 50%, although did not meet the clinical endpoint of reducing neuropathic pain [87]. AL1211 is the newest SRT with phase 2 clinical trials ongoing in men with classical FD [88]. Clearly these therapies show promise, but larger scale studies into their efficacy in reducing important clinical endpoints are needed.

Genetic substrate reduction therapy (gSRT) is a newer approach that has evolved from the concept of SRT. Rather than small-molecule inhibition of target enzymes in conventional SRT, gSRT involves selectively downregulating genes implicated in the synthesis and subsequent accumulation of substrate in lysosomal storage disorders. Techniques under investigation include use of gene-silencing technology, for example, using interference RNA (iRNA). This technology achieved a notable milestone in 2018 when it was approved in the US in the management of transthyretin-mediated amyloidosis, with subsequent approval by the National Institute for Health and Care Excellence for use in the UK [89,90]. In vitro studies have demonstrated that silencing of Gb3 synthase mRNA is possible using small interfering RNA (siRNA) [91,92]. Further work has explored whether suppression of the Gb3 synthase gene affected the disease phenotype of a model of FD nephropathy in vitro, by using induced pluripotent stem cells to develop a kidney organoid system. Knockout of the relevant gene using gene-editing technology led to improved α-GalA activity and reduced markers of renal damage [93]. This technology has shown encouraging results in pre-clinical studies; however, it has not yet been applied in vivo.

As opposed to gSRT, which reduces Gb3 production by silencing synthesis-related genes, other gene therapies under investigation have aimed to restore α-GalA activity. This may be achieved by delivering synthetic mRNA encoding α-GalA into cells, allowing them to produce the enzyme, or by introducing a functional copy of the GLA gene permanently using viral vectors. The latter would allow patients to receive a single treatment, negating the need for repeated enzyme infusions. Commonly used viral vectors for delivery of gene therapy include adeno-associated virus, adenovirus, and lentivirus. In a pilot safety study, five males with classical FD received lentivirus-transduced hematopoietic stems cells engineered to express α-GalA. The treatment was well tolerated, and all patients produced sufficient α-GalA at one week [94]. Early clinical trials are ongoing, and preliminary results suggest stabilization of renal function and improved patient-reported outcomes [95]. mRNA therapies that restore expression of the GLA gene have been investigated in vitro using induced pluripotent stem cell from patients with FD, differentiated to cardiomyocytes. α-GalA enzyme activity was sufficiently restored, and accumulation of Gb3 was reduced [96]. Further clinical trials are needed to determine the clinical efficacy and safety of gene therapies.

Disease-modifying treatments including ERT, OCT, substrate reduction therapy, and gene therapy aim to target the underlying pathophysiology of Gb3 accumulation and subsequent cell damage, but evidence for long-term efficacy is poor, limited to few small studies, and these treatments are costly. As previously discussed, there is probably a point beyond which simple storage is less important than the molecular cascades that are triggered in the process of accumulation, leading to cellular dysfunction and chronic inflammation, which drives end organ damage. Targeting these pathways with adjunctive treatments in a multi-faceted approach may prove fruitful in improving cardiovascular and renal outcomes in patients with FD.

## 5. Novel Therapies

Traditionally, metabolic risk, cardiovascular disease and renal complications are treated in isolation. Recent insights suggest that these systems are interconnected, and a paradigm shift is needed to move beyond an organ-focused approach, to an integrated multispecialty approach to therapeutics in cardiometabolic disease [97,98]. Novel therapies, including sodium-glucose cotransporter-2 (SGLT2) inhibitors, glucagon-like peptide-1 (GLP-1) agonists, and mineralocorticoid receptor antagonists (MRAs) shown to be effective in conditions such as type 2 diabetes (T2DM), heart failure, and CKD, and it is likely these drugs target shared pathways implicated in metabolic, cardiovascular, and renal disease. This is particularly relevant to FD, as insights into pathophysiology have implicated multi-system inflammation, fibrosis, and dysregulation of metabolism as key contributors to disease progression. This section explores the evidence supporting these therapies, mechanisms of action, and the potential application in the management of FD.

### 5.1. SGLT2 Inhibitors

SGLT2 inhibitors, originally developed for the management of T2DM due to their antihyperglycemic action, have shown significant cardiovascular and renal benefits extending beyond their impact on glycemic control. Landmark trials have demonstrated that these benefits also apply to patients without diabetes, prompting broader investigation into their cardioprotective and renoprotective effects. While the precise mechanisms driving these effects remain an active area of research, influence on haemodynamics as well as key cellular pathways and suppression of inflammatory processes are thought to play a significant role. Given the prevalent cardiac and renal involvement in FD, this drug class may offer valuable therapeutic benefits despite a lack of direct studies in FD populations.

SGLT2 inhibitors reduce reabsorption of sodium and glucose in the proximal tubule of the kidneys and increase glucose excretion in the urine. The increased distal tubular sodium load inhibits the renin-angiotensin-aldosterone system, reducing both preload and afterload, wielding its cardioprotective effects [99]. Similarly, the increased distal sodium load is renoprotective, as it reduces intra-glomerular pressure and albuminuria by tubuloglomerular feedback mechanisms [100]. These mechanisms are particularly relevant in FD, where renal dysfunction and progressive cardiomyopathy are key disease manifestations. Beyond hemodynamic effects, SGLT2 inhibitors directly influence cellular metabolism and inflammation, pathways that are dysregulated in FD. In the mitochondria, SGLT2 inhibitors have been shown in vivo to modulate the mTOR/AMPK pathway of autophagy, a process essential for cellular functioning. Dysregulated autophagy via this pathway has been demonstrated in podocyte cell models of FD, which may contribute to progressive renal dysfunction [47]. This could be a promising therapeutic target. In animal models of T2DM, empagliflozin enhances autophagy, preserves cardiac microvascular function, and suppresses mitochondrial ROS via its effects on the mTOR/AMPK pathway [101,102]. SGLT2 inhibitors also exert significant anti-inflammatory effects, which could counteract the chronic inflammation seen in FD. They reduce expression of inflammatory mediators, including TNF-α, IL-1β, IL-6, ICAM-1, PECAM-1, MMP2, MMP9, and ROS [103,104], and promote a shift from the pro-inflammatory M1 macrophage subtype to the anti-inflammatory M2 type [105]. In vitro, dapagliflozin also inhibits the expression of TLR-4 and NF-κB, suppressing secretion of the pro-inflammatory cytokines IL-1β, IL-6, IL-8, and TNF-α [105]. Since TLR-4 and NF-κB activation are triggered by accumulated lyso-Gb3 in FD [49], this highlights a mechanistic link between SGLT2 inhibitors and potential disease modification in FD, making them a compelling target for further research in Fabry-related cardiomyopathy and nephropathy.

Clinical trials have consistently demonstrated the benefits of SGLT2 inhibitors in improving cardiovascular and renal outcomes, irrespective of diabetes status. Given that FD is a multisystem disorder with predominant cardiac and renal complications, these findings support the potential therapeutic role of SGLT2 inhibitors. The DAPA-HF and EMPEROR-Reduced trials were pivotal in demonstrating the cardiovascular benefits of SGLT2 inhibitors in terms of reduced heart failure hospitalization and cardiovascular death in patients with heart failure with reduced ejection fraction (HFrEF), regardless of diabetes status [106,107]. Both trials enrolled patients with HFrEF (ejection fraction < 40%), NYHA class II-IV, who were receiving standard heart failure therapy and with a GFR > 30 mL/min/1.73 m^2^. Both dapagliflozin and empagliflozin reduced a composite outcome of heart failure hospitalization or cardiovascular death. Although patients with FD may develop HFrEF in advanced cardiomyopathy, HFpEF is the predominant cardiac phenotype characterized by progressive LVH, diastolic dysfunction, and fibrosis. The DELIVER and EMPEROR-preserved trials demonstrated similar benefits of SGLT2 inhibitors in patients with HFpEF [108,109]. Patients with chronic heart failure and with LVEF > 40%, elevated NT-proBNP, and NYHA class II-IV symptoms were enrolled. The DELIVER trial showed an 18% reduction in heart failure hospitalization or cardiovascular death with dapagliflozin, and empagliflozin again showed significant reduction in this composite outcome. Results from a recent meta-analysis also demonstrated the time-varying effects of SGLT2 inhibitors, in that reduction in endpoints such as HF hospitalization and all-cause mortality were greatest within 3 months of initiating therapy [110].

Whilst the DAPA-HF, DELIVER, and EMPEROR trials included patients with mild-moderate renal impairment (mean GFR 60–70 mL/min/1.73 m^2^), the DAPA-CKD and EMPA-KIDNEY trials explored the use of SGLT2 inhibitors in patients with more severe renal impairment. This is particularly relevant in FD where progressive renal decline is an important complication. Dapagliflozin and empagliflozin again reduced risk of progression of renal disease (defined by degree of decline in GFR) and associated complications, irrespective of diabetes status [111,112]. Few studies to date have investigated SGLT2 inhibitors in patients with FD specifically; however, one small case-series of 11 patients demonstrated that SGLT2 inhibitors successfully reduced albuminuria [113]. The results of a multicenter prospective cohort study exploring the impact of dapagliflozin on albuminuria, renal, and cardiac disease progression and exercise capacity are eagerly anticipated [114]. Extrapolation of the available evidence suggests that SGLT2 inhibitors may also offer therapeutic benefits in advanced Fabry nephropathy.

Evidence from these landmark trials supports the potential application of SGLT2 inhibitors in FD. The effects of SGLT inhibitors appear to be consistent, regardless of diabetes status or heart failure phenotype, with trials also demonstrating efficacy across the spectrum of renal dysfunction, including those with a GFR < 30 mL/min/1.73 m^2^. Given that FD is a multisystem disorder primarily involving cardiac and renal complications, SGLT2 inhibitors address both key issues. The promising effects of SGLT2 inhibitors in patients with symptomatic HFpEF are particularly relevant, as only in the advanced stages of disease do patients develop systolic impairment, by which point, prognosis is poor. Participants in landmark trials shared co-morbidities prevalent in the FD population, such as hypertension, atrial fibrillation (AF), T2DM, and high BMI. In keeping with trial data, SGLT2 inhibitors may be considered in patients with FD who have symptomatic HFpEF (NYHA class II–IV), and this includes patients with advanced cardiomyopathy who may not be eligible for ERT. Progressive renal impairment is another hallmark of FD, and while there is evidence that ERT slows GFR decline to some extent, additional therapies are needed. Like ERT, earlier treatment with SGLT2 inhibitors is probably better, and meta-analysis data support the urgency of initiating such evidence-based therapies, with most of the benefit achieved within 3 months [110]. Although specific studies in FD are lacking, the generalizability of trial findings and the robust theoretical basis for their mechanism of action support the use of SGLT2 inhibitors in FD.

### 5.2. GLP-1 Agonists

GLP-1 agonists are emerging as a promising therapy in a range of cardiovascular diseases. Originally developed primarily for metabolic conditions including T2DM and obesity, evidence from cardiovascular and renal outcome studies indicates significant benefits that extend beyond the effects of weight loss and improved metabolic profiles. This further emphasizes the concept that the pathophysiology of metabolic, cardiovascular, and renal disease is closely interrelated and should be managed holistically [97,98]. This is precisely why GLP-1 agonists are so promising, as their beneficial effects cross boundaries, targeting shared inflammatory, metabolic, and vascular pathways that are also integral to the pathophysiology of FD.

GLP-1 agonists act in the pancreas to increase synthesis and release of insulin by increasing neogenesis, proliferation, and reducing apoptosis of pancreatic β cells [115]. Beyond their role in glucose metabolism, these agents also modulate key inflammatory pathways implicated in FD, similar to SGLT2 inhibitors. GLP-1 agonists activate the AMPK pathway which negatively modulates NF-κB via the mTOR signaling pathway [116]. NF-κB is an important transcription factor driving M1 pro-inflammatory macrophage activation and the release of pro-inflammatory cytokines [117,118], both of which are implicated in chronic inflammation and endothelial dysfunction in FD [49,52]. In vivo, the NF-κB signaling pathway has been implicated in the development of cardiac hypertrophy, a hallmark of Fabry cardiomyopathy [119]. In cardiac fibrosis mice models, GLP-1 agonists reduced fibroblast and myofibroblast expression in cardiac myocytes, also inhibiting cardiac myocyte hypertrophy [120]. Another in vivo study demonstrated the GLP-1 agonist exendin-4 reduced macrophage infiltration, suppressed pro-inflammatory cytokines, and protected against cardiac remodeling and diastolic dysfunction. These anti-inflammatory effects extend to the kidneys, where GLP-1 agonists directly inhibit TNF-α–mediated NF-κB activation in podocytes, suggesting a potential renoprotective role in Fabry nephropathy [121,122]. Beyond cellular signaling, GLP-1 agonists also exhibit natriuretic effects, and this mechanism is thought to be responsible at least in part for blood pressure reductions seen in major clinical trials, again emphasizing the molecular benefits of these medications beyond the effects of weight loss alone [123]. Given that hypertension accelerates renal and cardiovascular complications in FD, this effect may offer additional therapeutic benefit. Furthermore, GLP-1 agonists have been shown to have direct anti-atherosclerotic effects in euglycemic mice and hyperlipidemic rabbits [124,125]. Conventional risk factors for atherosclerosis and coronary artery disease are prevalent in patients with FD [126,127]. Additionally, FD-specific mechanisms, including nitric oxide dysregulation, oxidative stress, and microvascular endothelial dysfunction secondary to sphingolipid accumulation, are thought to accelerate vascular disease progression [57]. By these molecular mechanisms, it is proposed that GLP-1 agonists may improve cardiovascular outcomes in patients with FD by targeting underlying mechanisms of inflammation, hypertrophy, and fibrosis.

In cardiovascular and renal outcome trials, GLP-1 agonists have consistently shown reductions in major adverse cardiovascular outcomes (MACE) and reduced risk of renal failure in patients with T2DM [128,129,130,131]. More recent trials demonstrate cardiovascular benefits in non-diabetics, including those who are overweight or obese, improving blood pressure, cholesterol, and triglyceride levels, and reducing cardiovascular events [132,133,134]. Given that FD is associated with a high burden of cardiovascular and renal complications, these findings may have important implications for FD patients, even in the absence of diabetes.

The SELECT trial evaluated the effects of semaglutide on MACE in adults with a BMI ≥ 27 and established cardiovascular disease (defined as previous myocardial infarction, stroke, or symptomatic peripheral arterial disease) without diabetes. This is in comparison to other key trials that primarily focused on patients with diabetes, and where weight loss was the primary outcome. The SELECT trial demonstrated a significant reduction in the composite outcome of MACE in the semaglutide arm, with similar reductions in blood pressure and improvement in lipid profiles as seen in the earlier STEP and SCALE trials [135]. GLP-1 agonists have also demonstrated benefits in patients with HFpEF; this is especially relevant in FD where HFpEF is the predominant cardiac phenotype. In a pre-specified subgroup analysis of over 4000 patients in the SELECT trial with heart failure, semaglutide reduced composite heart failure endpoints in those with HFrEF and HFpEF, independent of age, sex, and baseline BMI [136]. Furthermore, there were improvements in symptoms and exercise capacity in patients with HFpEF [137], which is of major significance, as even early-stage cardiomyopathy has been shown to impair functional capacity in FD [25]. This was supported by pooled analysis of trials of GLP-1 agonists in HFpEF from the SELECT, FLOW, STEP-HFpEF, and STEP-HFpEF DM trials, which showed that semaglutide significantly improved clinical symptoms, physical limitations, and 6 min walk distance [138]. In the SELECT trial, differences in primary and secondary endpoints emerged early, and it is proposed that early treatment-related physiological changes independent of the magnitude of weight loss may have contributed to the improved cardiovascular outcomes [135]. The FLOW trial investigated semaglutide in patients with CKD and T2DM. Semaglutide reduced the risk of major kidney disease events (composite of renal failure, 50% reduction in eGFR from baseline or death from kidney or cardiovascular causes) by 24% [131]. Subgroup analysis of the FLOW trial including patients with both CKD and HF also demonstrated a reduction in time to first HF event and cardiovascular death [139].

Tirzepatide, a dual glucose-dependent insulinotropic polypeptide (GIP) and GLP-1 agonist, offers synergistic metabolic benefits proven superior to GLP-1 agonists alone. Clinical trials have shown superior weight loss and glycemic control compared to standard GLP-1 agonists (SURMOUNT-1, SURPASS-2 trials) [140,141]. A large cohort study also showed that tirzepatide was superior to GLP-1 agonists in terms of all-cause mortality, MACE, and major adverse kidney events in patients with diabetes [142]. The SUMMIT trial investigated tirzepatide in patients with HFpEF and obesity (BMI ≥ 30 kg/m^2^) with or without T2DM, demonstrating a 38% reduction in cardiovascular death worsening HF events over 12 months of follow-up [143].

The indirect benefits of GLP-1 agonists and GIP/GLP1-agonists on cardiovascular and renal risk factors including weight, blood pressure, and lipid profiles are well established; however, their ability to modulate inflammation, fibrosis, and vascular dysfunction through pathways shared with FD highlights their potential as a promising therapeutic avenue for Fabry cardiomyopathy.

In FD specifically, GLP-1 agonists may be used for conventional indications such as T2DM or for weight management; however, there is a strong argument that use should be broadened based on its proven cardiovascular and renal benefits. The evidence for GLP-1 agonists in symptomatic HFpEF is of particular interest in FD. Although the SELECT trial exclusively enrolled individuals with atherosclerotic cardiovascular disease, the relevance to FD remains significant, as atherosclerosis often co-exists in patients with FD due to conventional risk factors and disease-specific mechanisms. The safety and efficacy of GLP-1 agonists in more advanced renal disease was explored by the FLOW trial, with promising results [131]. The mechanisms demonstrated in preclinical and clinical studies, combined with evidence from key clinical trials, suggest GLP-1 agonists and GIP/GLP-1 agonists could address both conventional cardiovascular risk factors and FD-specific pathophysiology. Trials specific to FD are essential to validate these potential benefits, and future trials should consider integrated endpoints rather than focusing solely on metabolic, cardiovascular, and renal outcomes independently given the complex interplay.

### 5.3. Mineralocorticoid Antagonists

Mineralocorticoid receptor antagonists (MRAs) are one of the pillars of heart failure therapy. This class of drug includes spironolactone and eplerenone, both steroidal MRAs, as well as the newer non-steroidal agent, finerenone. Emerging trial data suggest that finerenone may offer an improved efficacy and safety profile compared to traditional steroidal MRAs, proposed to be due to its unique pharmacological properties. Finerenone has greater selectivity and greater receptor binding affinity and distributes equally in tissue between the heart and kidney, unlike steroidal MRAs [144]. It is thought that the anti-inflammatory and anti-fibrotic effects of finerenone may contribute to observed benefits and align closely with pathological mechanisms of Fabry cardiomyopathy and nephropathy.

The pharmacological rationale for MRAs lies in the understanding that overactivation of mineralocorticoid receptors in various tissues contributes to tissue injury, driving inflammation and fibrosis in the cardiovascular and renal systems. Mineralocorticoid receptor stimulation promotes the activation of various inflammatory pathways and cytokine release. The aldosterone/mineralocorticoid receptor complex regulates expression of enzymes responsible for production of ROS and oxidative stress, inhibition of which with MRAs have been shown to reduce oxidative stress in mice [145]. Oxidative stress activates the NF-κB signaling pathway, which plays an important role in inflammation and fibrosis via the induction of pro-inflammatory cytokines including IL-6, TNF-α, and IFN-γ [146,147]. Given that NF-κB activation is a key driver of Fabry-related inflammation and fibrosis [49], MRAs may help mitigate these pathological effects in FD patients. Additionally, mineralocorticoid receptors are expressed on immune cells, and their activation induces M1 macrophage polarization, a contributor to FD-related inflammation. In renal injury induced in mice, finerenone promoted increased IL-4 receptor expression, which facilitated polarization of macrophages to the M2 anti-inflammatory type [148]. In addition to their anti-inflammatory effects, MRAs also exhibit antifibrotic effects, which is critical in Fabry cardiomyopathy and nephropathy characterized by progressive organ fibrosis. In mouse models where renal fibrosis was induced, finerenone attenuated pathological myofibroblast activation, collagen deposition, and expression of kidney plasminogen activator inhibitor-1 (PAI-1) and naked cuticle 2 (NKD2), key mediators of tissue fibrosis [149]. In a cardiac model of isoproterenol-treated mice, improvement of global longitudinal strain on speckle tracking echocardiography was seen with finerenone, and to a lesser extent, eplerenone [150]. Whilst both finerenone and eplerenone reduced LV mass, only finerenone reduced macrophage invasion and development of cardiac fibrosis. At a molecular level, treatment with finerenone was associated with inhibition of profibrotic cardiac *TNX* (*tenascin-X*) expression, an effect not seen with eplerenone and one that may explain the enhanced antifibrotic effects of finerenone compared with other MRAs [150]. Other profibrotic markers, including connective tissue growth factor (CTGF), lysyl oxidase (LOX), and TGF-β, are also attenuated by finerenone [151], further reinforcing its potential to mitigate fibrotic remodeling in FD.

The FINEARTS-HF trial investigated finerenone versus placebo in patients with symptomatic HFpEF (EF > 40%) in over 6000 patients. Finerenone reduced the composite outcome of worsening heart failure events and death from cardiovascular causes, contributed to mostly by reduction in heart failure events. Around half of included patients had co-existing renal impairment (GFR < 60 mL/min/1.73 m^2^) and over 60% had been hospitalized for heart failure previously [152], a study population that closely reflects the clinical burden seen in patients with Fabry cardiomyopathy and nephropathy. The FIDELIO-DKD and FIGARO-DKD trials investigated finerenone versus placebo in patients with CKD and T2DM. Most included patients had at least moderate renal impairment at baseline (GFR 25–60 mL/min/1.73 m^2^) and significant proteinuria (ACR ≥ 300 mg/g). Finerenone reduced the composite cardiovascular outcome of death from cardiovascular causes, non-fatal myocardial infarction, non-fatal stroke, and hospitalization for heart failure by 13%, again contributed to mostly by the reduction in incidence of heart failure events [153]. Finerenone also improved renal outcomes including reduced progression of renal failure and death from renal causes [154]. Emerging evidence also suggests that finerenone has a better safety profile compared with steroidal MRAs in terms of risk of hyperkalemia, worsening GFR, androgenic side effects, with similar efficacy; however, head-to-head trials with longer term outcome data are needed [155].

The underlying anti-inflammatory and anti-fibrotic properties of finerenone are of particular interest in cardiovascular diseases driven by inflammation and fibrosis, such as FD. Trial data suggest that finerenone improves cardiovascular outcomes in patients with symptomatic HFpEF and co-existing CKD, a phenotype common to the FD population. Finerenone also improves renal outcomes, although only studied in the context of CKD caused by diabetic nephropathy, renal manifestations of FD are thought to progress at a similar rate, with shared structural changes at the nephron as in diabetic nephropathy [5]. MRAs, and finerenone in particular, should be considered in the management of HFpEF and renal dysfunction in FD.

### 5.4. IL-6 Inhibitors

IL-6 inhibitors are an emerging class of drug with mechanisms of interest in FD. IL-6 is an interleukin implicated in pro-inflammatory inflammatory signaling and immune regulation. IL-6 knockout mice shown reduced cardiac remodeling dysfunction, reduced M1 macrophage, and increased M2 macrophage differentiation [156]. High levels of the cytokine IL-6 have been associated with worse cardiovascular outcomes and mortality in patients with cardiovascular disease [157,158]. As previously outlined, IL-6 has consistently been shown to be implicated in mechanistic models of FD. IL-6 inhibitors are well established as a therapeutic option in certain inflammatory and autoimmune conditions such as rheumatoid arthritis, systemic juvenile idiopathic arthritis, and COVID-19. They have more recently been applied in cardiovascular diseases as understanding of pathophysiology improves. The RESCUE phase 2 clinical trial showed that intravenous ziltivekimab, an IL-6 inhibitor, reduced biomarkers of inflammation and thrombosis relevant to atherosclerotic disease in patients with renal failure [159]. Similar results were shown with clazakizumab [160]. In acute ST elevation myocardial infarction, tocilizumab increased myocardial salvage with less microvascular obstruction [161]. This new class of drug may be of promise in FD and clinical trials in the FD population are needed.

### 5.5. Colchicine

Colchicine, an anti-inflammatory drug primarily used to treat gout and pericarditis, has become a drug of interest in a spectrum of cardiovascular diseases due to its direct effects on the inflammatory cascade. Colchicine exerts its anti-inflammatory effects by preventing migration and adhesion of neutrophils and suppressing the NLRP3 inflammasome, decreasing IL-1β and IL-6 production, cytokines implicated in the pathophysiology of FD [162]. Additionally, colchicine is thought to exhibit anti-fibrotic effects and has been shown to suppress fibrosis in a mouse-models of renal disease [163].

The cardioprotective effects of colchicine have been supported in key trials such as the COLCOT trial, investigating colchicine post acute myocardial infarction, demonstrating a reduction in ischemic cardiovascular events [164]. Similar benefits were seen in patients with chronic coronary disease [165]. However, in patients with HFrEF, colchicine did not reduce progression of heart failure or improve symptoms [166]. As a cost-effective alternative to monoclonal antibodies inhabiting specific interleukins like IL-6, colchicine may warrant further exploration in FD specifically, although potential use may be constrained by gastrointestinal intolerance, already a common issue in FD.

### 5.6. Future Directions

Current medications on the market, such as SGLT2 inhibitors, GLP-1 agonists, and non-steroidal MRAs, address key drivers of cellular dysfunction and inflammation. Although their mechanisms are broad, these drugs address pathways implicated in the pathophysiology of FD. They have demonstrated significant cardiovascular and renal benefits in conditions like HFpEF, HFrEF, and CKD, but their potential in FD remains unexplored. Further research is needed to assess the clinical efficacy in the FD population, particularly in early-stage, asymptomatic disease where therapeutic intervention may be most impactful. Additionally, these drugs should be investigated in advanced disease, where treatment options are limited due to current ERT initiation guidelines. Trials must include diverse FD phenotypes, such as females and those with non-classical disease, to ensure findings are broadly applicable. Research should also focus on the drugs’ ability to modulate FD-specific pathways in vivo, alongside the development of novel FD-specific biomarkers to monitor treatment efficacy and enable personalized therapy. Advancing research in this area will expand treatment options available to patients with FD, akin to the ‘four-pillar’ approach in heart failure [167], cumulative benefits of these widely used drugs may transform care for this complex, multi-system disorder.

## 6. Conclusions

A growing perspective on the pathophysiology of FD is that it extends beyond being merely a storage disorder, which likely accounts for the limited efficacy of ERT observed in clinical trials. Evidence suggests there is a threshold beyond which ERT loses its effectiveness, allowing organ damage and dysfunction to progress despite treatment. As our understanding of the disease’s pathophysiology and the mechanism behind cellular dysfunction deepens, it is evident that therapies must target the unrelenting cascade of mitochondrial dysfunction, endothelial damage, and inflammation that ensues, triggered by glycosphingolipid accumulation. Numerous widely available adjuvant treatments with a solid theoretical foundation for targeting these pathways have been extensively studied in both cardiovascular and non-cardiovascular conditions; however, further research specifically tailored to the FD population is essential. Novel therapeutics directly targeting key inflammatory pathways, such as IL-6 inhibitors and colchicine, are also of interest, but are yet to be investigated in cardiomyopathy.

## Abbreviations

The following abbreviations are used in this manuscript:

FD	Fabry disease
ERT	Enzyme replacement therapy
OCT	Oral chaperone therapy
SGLT2	Sodium-glucose cotransporter-2
GLP-1	Glucagon-like peptide-1
MRA	Mineralocorticoid antagonist
HFrEF	Heart failure with reduced ejection fraction
HFpEF	Heart failure with preserved ejection fraction
CKD	Chronic kidney disease
T2DM	Type 2 diabetes mellitus
AF	Atrial fibrillation
LV	Left ventricular hypertrophy
LGE	Late gadolinium enhancement
